# Influence of Neutralization Precipitation Conditions on the Physical Stability of Amorphous Solid Pharmaceuticals

**DOI:** 10.3390/molecules30040764

**Published:** 2025-02-07

**Authors:** Zhihui Yuan, Xu Liao, Bowen Zhang, Asad Nawaz, Zunhua Li

**Affiliations:** 1 College of Chemistry and Bioengineering, Hunan University of Science and Engineering, Yongzhou 425199, China; zhh_yuan@126.com (Z.Y.); liaoxuhuse@126.com (X.L.); 007298@yzu.edu.cn (A.N.); 2 School of Chemical Engineering and Technology, Tianjin University, Tianjin 300072, China; bowen_667@tju.edu.cn

**Keywords:** amorphous form, neutralization precipitation, physical stability, accelerated stability test

## Abstract

This research focused on the precipitation of amorphous forms of nilotinib with high physical stability through the manipulation of various parameters in the neutralization reaction, specifically the quantity of nilotinib, the pH value, and the concentration of HCl. To assess the physical stability of the amorphous nilotinib, various characterization techniques, including PXRD, DSC, and FBRM, were utilized in conjunction with analytical methods such as PDF, PCA, and R_c_ value. The findings demonstrated that the ideal physical stability was attained with a nilotinib quantity of 0.5 g, a pH value of 11.70, and 7.5 mL of HCl with a concentration of 2.0 mol/L. It is important to acknowledge that this observation is specific to the current experimental configuration and may not hold in the context of a scaled-up experiment. Furthermore, the combination of PDF and R_c_ was identified as an innovative and effective method for assessing physical stability, demonstrating advantages over traditional accelerated stability testing approaches.

## 1. Introduction

Due to most candidate compounds belonging to Class II or Class IV in the biopharmaceutical classification system (BCS), their low water solubility increases the difficulty of drug development. Low water solubility or low dissolution rates can lead to low drug bioavailability, severely affecting the clinical efficacy of the pharmaceuticals [1,2]. To address this trend, evaluating methods to improve oral bioavailability has become a routine process early in formulation development plans. In this context, amorphous solids have become one of the preferred technologies for enhancing bioavailability [3,4,5,6].

Amorphous forms exhibit unique characteristics due to their disordered state compared to crystal forms [7]. For example, the apparent water solubility of amorphous substances is significantly higher than that of crystal forms [8]. Additionally, due to the structural disorder of amorphous substances, their mechanical properties differ markedly from those of crystal states. Experiments have shown that under certain conditions, tablets formed from amorphous drug powders are more brittle compared to those from crystal states [9]. Furthermore, amorphous substances have a very strong hygroscopicity, absorbing more moisture than crystal forms in normal environments [10].

Amorphous pharmaceuticals, characterized by elevated surface free energy, are prone to transitioning into a crystal state, which can result in the loss of their formulation benefits [11]. Pharmaceutical manufacturing processes may inadvertently or deliberately produce amorphous forms that can have detrimental effects. Consequently, research into amorphous substances is of paramount importance. The process of amorphization exerts a considerable impact on the physical stability of amorphous solids [12,13,14,15,16,17,18,19,20], with factors related to anti-solvent precipitation being particularly critical [18,19,20]. Consequently, it is imperative to attain a thorough comprehension and regulation of these parameters to guarantee the stability and effectiveness of amorphous pharmaceutical formulations.

Traditionally, the physical stability of crystal solids has been evaluated through powder X-ray diffraction (PXRD). In contrast, the characterization of amorphous solids using PXRD encounters considerable difficulties, primarily due to the emergence of a halo peak [21]. Recent research indicated that the transformation of PXRD data into pair distribution function (PDF) format, coupled with the application of principal component analysis (PCA), serves as an effective method for evaluating the physical stability of amorphous solids [18,19,20,22]. In addition, the R_c_ indicator serves as a measure of amorphous solid stability [18,19,20,23]. In the present investigation, nilotinib, a compound characterized by limited solubility that adversely affects its bioavailability [24,25], was selected as the model substance. To overcome these solubility challenges, various formulation strategies have been studied [26,27,28]. The primary aim of this investigation was to induce the precipitation of amorphous nilotinib by controlling the quantities of nilotinib, pH values, and HCl concentration. The resulting solids were characterized through PDF analysis and R_c_ value assessment, while PCA was utilized to determine the optimal neutralization precipitation conditions that would enhance the physical stability of the amorphous solid pharmaceuticals.

## 2. Results and Discussion

### 2.1. Effect of Quantity of Nilotinib

#### 2.1.1. PDF and PCA of PXRD

**Figure 1:**
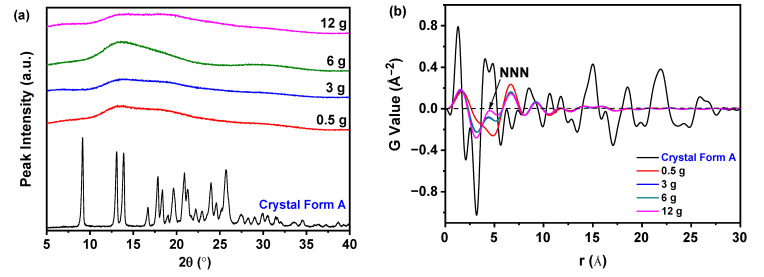

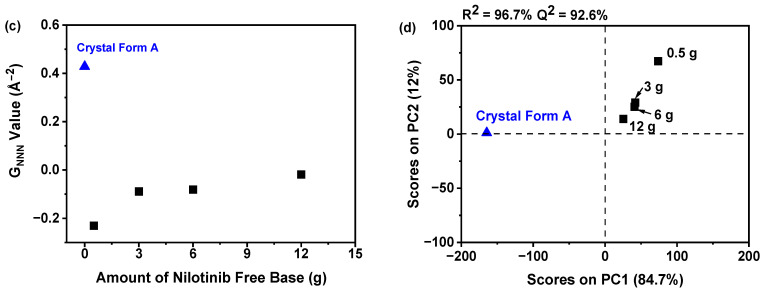
PDF and PCA of PXRD during the investigation of the quantity of nilotinib: (**a**) PXRD; (**b**) PDF; (**c**) G_NNN_; (**d**) PCA (The pH value and HCl concentration were fixed at 11.70 and 2.0 mol/L, respectively).

Figure 1a illustrates the influence of varying quantities of nilotinib on the structural properties of dried samples, all of which display halo peaks in PXRD analysis, indicative of amorphous characteristics, in contrast to the crystal Form A. It is important to note that differentiating between amorphous solids solely through PXRD is a complex task [29,30]. Consequently, PDF analysis was employed (Figure 1b), which identified a prominent peak at r = 4.62 Å. This peak serves as an indication of the next nearest neighbor (NNN) interactions associated with the lattice height of the crystal Form A of nilotinib [22,31]. This finding is consistent with the results reported by Yuan et al. [22] and bears resemblance to the analysis conducted on indomethacin [32]. The PDF has demonstrated significant efficacy in the structural analysis of amorphous pharmaceuticals, as evidenced by various studies [33,34]. It provides valuable insights into the solid-state order of these materials. For instance, PDF data about the polymorph of indomethacin revealed alterations in local structure during the grinding process [35]. Notably, the intensities of ordered crystal peaks were found to be greater than those of their amorphous counterparts [36]. Furthermore, Davis et al. employed PDF to quantify phase fractions and evaluate disorder in cryo-milled sulfamethazine [37]. In conclusion, PDF serves as a powerful tool for characterizing amorphous pharmaceuticals, elucidating structural properties, phase fractions, and physical stability, particularly the duration of crystallization [38,39]. An increase in order within amorphous samples was associated with a heightened propensity for recrystallization and a reduction in stability. This understanding is instrumental in elucidating the effects of processing on structural properties and stability, which is essential for optimizing formulation and storage conditions.

Figure 1c illustrates that samples treated with 0.5 g of nilotinib exhibited the lowest G_NNN_ value (the G value at the NNN peak) in comparison to those treated with 3, 6, and 12 g, indicating a decrease in disorder in samples with increased nilotinib quantities. Additionally, the PCA conducted on the PDF data, as depicted in Figure 1d, classified the samples into four distinct groups: crystal Form A, samples treated with 0.5 g of nilotinib, samples treated with 3 and 6 g of nilotinib, and samples treated with 12 g of nilotinib. It is noteworthy that the samples treated with 0.5 g of nilotinib were significantly different from those treated with more than 3 g, while the sample treated with 12 g of nilotinib exhibited characteristics similar to crystal Form A along the PC1, indicating the greatest resemblance to the reference sample (crystal Form A) concerning molecular disorder. These results are consistent with previous studies [32,39].

An increase in the quantity of nilotinib results in a reduction in disordered amorphous solids, attributed to the extended contact time with the aqueous solution during the filtration process, which promotes molecular rearrangement [19,20]. However, the introduction of higher amounts of nilotinib may also lead to the incorporation of additional impurities, such as HCl and NaOH, which can disrupt the molecular organization and hasten the crystallization process. At lower solute concentrations, the presence of weaker intermolecular interactions tends to favor the formation of amorphous structures. In contrast, at elevated concentrations, these interactions are strengthened, which may result in the conversion of amorphous structures into more stable crystal forms or more ordered amorphous solids. Furthermore, uneven stirring associated with increased nilotinib quantities can create local concentration disparities, thereby influencing stability. In the present study, the application of 0.5 g of nilotinib was found to produce the most physically stable amorphous solid.

#### 2.1.2. R_c_ Analysis of DSC

**Figure 2:**
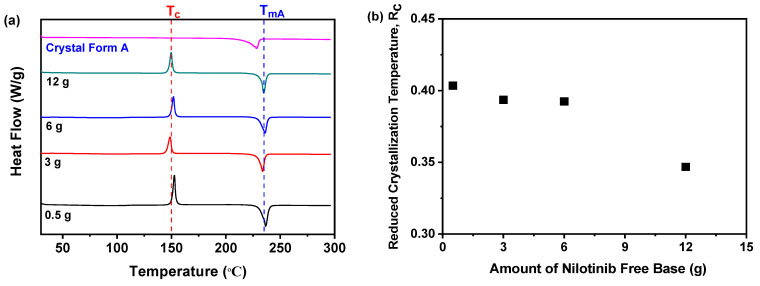
R_c_ analysis of DSC during the investigation of the quantity of nilotinib: (**a**) DSC; (**b**) R_c_ (The pH value and HCl concentration were fixed at 11.70 and 2.0 mol/L, respectively).

Differential Scanning Calorimetry (DSC) analyses confirmed the findings from PXRD studies. As illustrated in Figure 2a, all nilotinib samples exhibited endothermic peaks at 235 °C, which are indicative of the melting of crystal Form A [40,41]. Additionally, an exothermic peak observed at approximately 150 °C suggests a transition from amorphous to crystal states, consistent with established crystallization temperatures [18,19,20]. Additionally, elevated R_c_ values are associated with increased resistance to this transition, thereby contributing to enhanced physical stability. Consequently, as shown in Figure 2b, the R_c_ value demonstrated variability concerning the quantity of nilotinib. The sample treated with 0.5 g of nilotinib exhibited the highest R_c_ value, signifying superior physical stability. In contrast, samples treated with 3 g and 6 g of nilotinib displayed comparable R_c_ values, indicating that stability remained unaffected at these quantities. However, an increase in nilotinib from 6 g to 12 g resulted in a decrease in R_c_, suggesting a decline in stability. Consequently, it can be concluded that the optimal quantity of nilotinib for the production of amorphous solids with enhanced stability is 0.5 g.

#### 2.1.3. Filtering Rate Analysis

**Figure 3:**
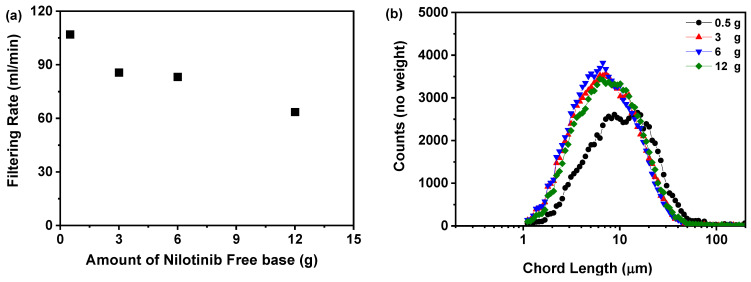

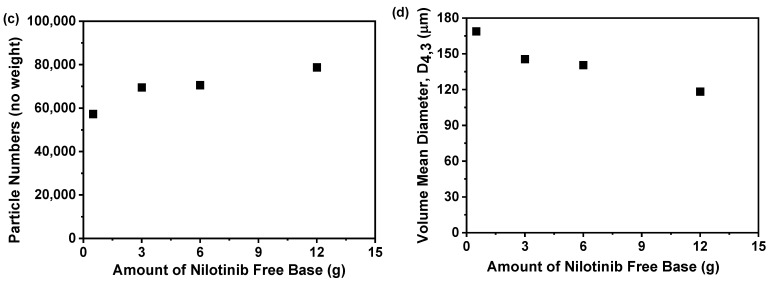
Filtering rate analysis during the investigation of the quantity of nilotinib: (**a**) filtering rate; (**b**) particle size distribution; (**c**) particle numbers; (**d**) volume mean diameter, D_4,3_ (The pH value and HCl concentration were fixed at 11.70 and 2.0 mol/L, respectively).

The data presented in Figure 3a demonstrate a clear inverse relationship between the quantity of nilotinib and the filtration rate. An increase in the quantity of nilotinib correlates with a reduction in the filtration rate, which decreases from 107 to 63.58 mL/min. This phenomenon is likely attributable to the formation of smaller solid particles that may obstruct the filtration medium, consequently impeding the filtration process. This observation is further corroborated by the data presented in Figure 3b–d. Figure 3b,c utilize focused beam reflectance measurement (FBRM) to confirm that a higher quantity of nilotinib leads to an increased particle count. This suggests that as more nilotinib is present, more particles are formed, potentially contributing to the slower filtration rates observed. Additionally, Figure 3d provides evidence of a direct relationship between the quantity of nilotinib and a decrease in particle size. As the nilotinib quantity increases, the volume mean diameter of the particles decreases from 168.87 to 118.35 μm, indicating that the particles are becoming finer. This reduction in particle size could explain the decline in filtration rate, as smaller particles are more likely to clog the filtration system. These findings are consistent with previous research [42], which has reported similar observations regarding particle size and filtration rates in different systems. Overall, the data presented provide valuable insights into the relationship between nilotinib quantity, particle size, and filtration rate.

Li et al.’s findings emphasize the importance of particle size and quantity in determining the duration of filtering, which is directly relevant to the present investigation [43]. To mitigate negative impacts on filtration rate and physical stability, the research constrained the duration of precipitation and storage to a maximum of 30 min before filtration. Extended filtration times may prolong the overall processing duration, which poses a risk of crystallization and may modify the physical characteristics of amorphous solids, consequently diminishing their efficacy. Therefore, by carefully controlling the precipitation and storage times, the study aimed to ensure that the filtration process was efficient and that the amorphous solid remained stable throughout the process. This approach is crucial for the development of robust and reliable manufacturing processes for amorphous solids, such as nilotinib, which have important therapeutic applications.

### 2.2. Effect of pH Value

#### 2.2.1. PDF and PCA of PXRD

**Figure 4:**
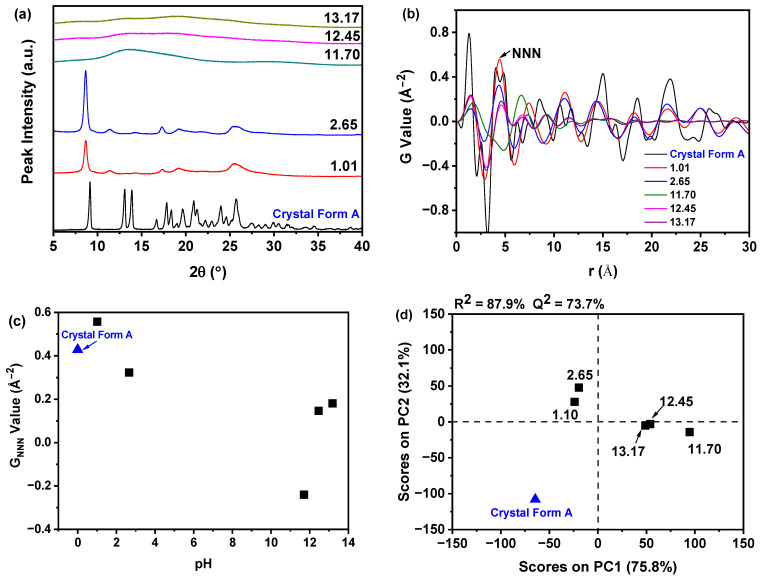
PDF and PCA of PXRD during the investigation of pH value: (**a**) PXRD; (**b**) PDF; (**c**) G_NNN_; (**d**) PCA (The quantity of nilotinib and HCl concentration were fixed at 0.5 g and 2.0 mol/L, respectively).

As illustrated in Figure 4a, the results indicate that alkaline conditions, specifically with pH values of 11.70, 12.45, and 13.17, led to the generation of nilotinib amorphous form. Conversely, samples synthesized under acidic conditions, with pH values of 1.01 and 2.65, yielded a distinct crystal form that is not congruent with crystal Form A of nilotinib. This crystal form has been unequivocally identified as nilotinib hydrochloride monohydrate, exhibiting characteristic diffraction peaks at 2θ angles of 8.66°, 11.42°, 17.37°, 19.23°, and 25.50°, which follow previously published data [44,45,46]. However, it is noteworthy that the PXRD patterns of the samples prepared at pH 1.01 and 2.65 exhibited a reduced number of peaks and decreased peak intensities compared to those reported in the literature, implying a lower degree of crystallinity within these samples. Furthermore, as depicted in Figure 4b, the samples prepared at pH values of 1.01 and 2.65 exhibited greater fluctuations in the NNN peak intensities compared to those prepared at other pH values. The observed variation in NNN peak height suggests that the pH value significantly influences the disorder within the samples. Specifically, as the pH value increased, the peak height of NNN initially decreased and subsequently increased. This pattern indicates that a pH value of 11.70 is conducive to the formation of a more physically stable amorphous solid, exhibiting a high degree of structural disorder.

As depicted in Figure 4c, the G_NNN_ values for samples synthesized at pH values of 1.01 and 2.65 were observed to surpass those of samples prepared at pH values of 11.70, 12.45, and 13.17. Notably, the sample synthesized at the optimal pH value of 11.70 exhibited the lowest G_NNN_ value, signifying the highest degree of structural disorder. Consequently, a pH of 11.70 was chosen for the current investigation. Furthermore, as demonstrated in Figure 4d, in comparison to the reference sample, the PCA score plot successfully classified all samples prepared at different pH values into four distinct clusters. The first cluster encompassed crystal Form A, while the second cluster comprised samples prepared at pH values of 1.01 and 2.65, which were proximal to crystal Form A, suggesting structural similarity. The third cluster consisted of samples prepared at pH values of 12.45 and 13.17, and the fourth cluster included the sample prepared at pH 11.70, which was the most distant from crystal Form A, indicating the greatest structural deviations from this reference form.

The p*K*_a_ value is a critical physicochemical characteristic of pharmaceuticals, significantly impacting various biopharmaceutical properties in pharmacokinetic and bioavailability assessments. This parameter indicates the deprotonation state of a molecule within a specific solvent. The dissociation constants of a compound are instrumental in determining its lipophilicity, solubility, and permeability, thereby playing an essential role in defining its absorption, distribution, metabolism, and excretion (ADME) profile [47,48,49,50,51]. Consequently, p*K*_a_ values are vital for both the analysis of pharmaceutical agents and the elucidation of their mechanisms of action. Meloun et al. have demonstrated that nilotinib hydrochloride, referred to as L, can form four distinct water-soluble cations: LH^+^, LH_2_^2+^, LH_3_^3+^, and LH_4_^4+^. The distribution diagram illustrating the relative concentrations of the variously protonated ions of nilotinib hydrochloride indicates that the species can be represented as follows: the anion L^−^ predominates at pH values between 11 and 14, the neutral molecule is prevalent at pH values from 6 to 11, the cation LH^+^ is dominant at pH values from 2 to 6, the cation LH_2_^2+^ is present at pH values from 0 to 3, and the cation LH_3_^3+^ is observed at pH values from 0 to 2 [52]. In the context of this study, at pH values of 1.10 and 2.65, the formation of cations LH_2_^2+^ and LH_3_^3+^ was noted, corresponding to the nilotinib hydrochloride monohydrate crystal solid form. Conversely, at pH values exceeding 11 (specifically at 11.70, 12.45, and 13.17), the formation of the anion L^−^ was observed, indicating the presence of the nilotinib amorphous form.

#### 2.2.2. R_c_ Analysis of DSC

**Figure 5:**
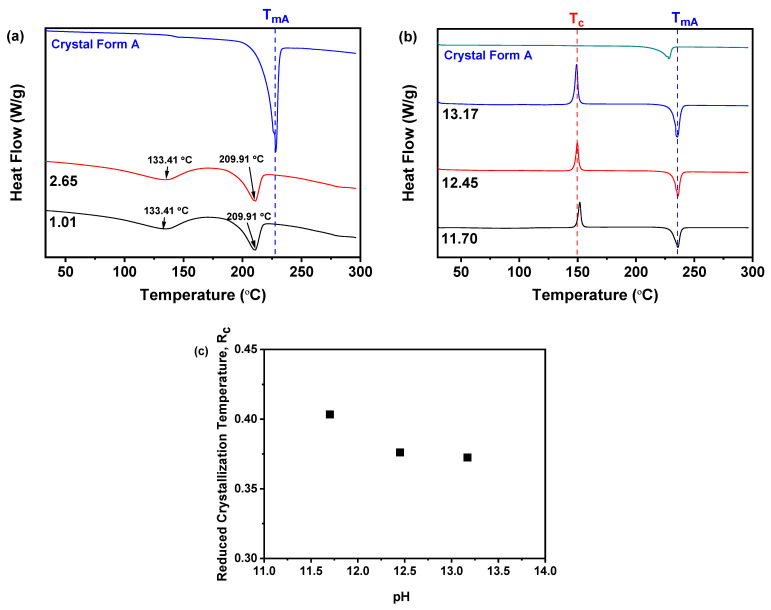
R_c_ analysis of DSC during the investigation of pH value: (**a**) DSC; (**b**) DSC; (**c**) R_c_ (The quantity of nilotinib and HCl concentration were fixed at 0.5 g and 2.0 mol/L, respectively).

As shown in Figure 5a, the samples obtained under acidic conditions at pH values of 1.01 and 2.65 exhibited two distinct endothermic peaks in their DSC profiles. The DSC curve for nilotinib hydrochloride monohydrate revealed an initial endothermic peak at T_peak_ = 133.41 °C attributed to the loss of water during heating. The subsequent endothermic peak at T_peak_ = 209.91 °C represented the melting temperature of nilotinib hydrochloride monohydrate. These observations suggest that the melting process is preceded by the conversion of nilotinib hydrochloride monohydrate to its anhydrous salt form, which is consistent with previous literature reports [45,46]. Conversely, as depicted in Figure 5b, the samples prepared under alkaline conditions at pH values of 11.70, 12.45, and 13.17 exhibited a unique endothermic peak corresponding to the T_mA_. Furthermore, Figure 5c indicated that the R_c_ was influenced by the pH value during synthesis. Among the three amorphous solids, the sample synthesized at a pH of 11.70 exhibited the highest R_c_ value. However, raising the pH value from 11.70 to 13.17 led to a decrease in the R_c_ value, suggesting a gradual decline in physical stability. Consequently, a pH value of 11.70 was identified as the optimal condition for precipitating physical stability of amorphous solids via the neutralization reaction in this study.

### 2.3. Effect of HCl Concentration

#### 2.3.1. PDF and PCA of PXRD

**Figure 6:**
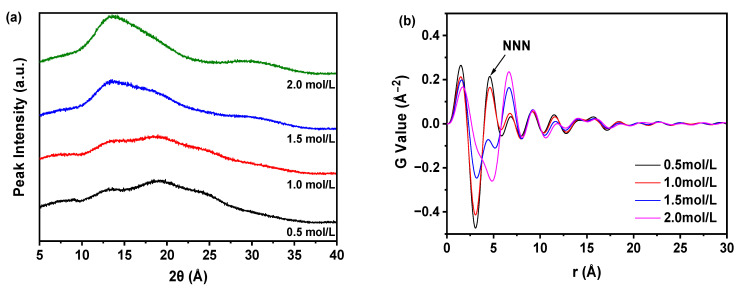

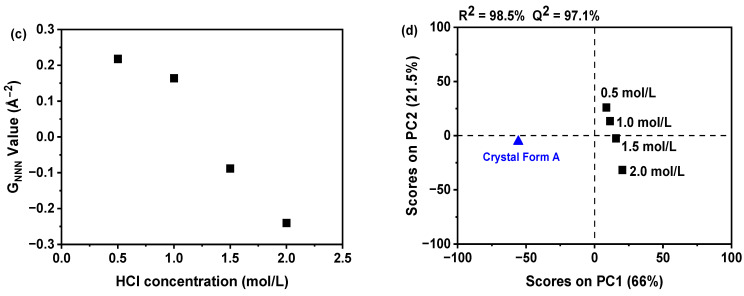
PDF and PCA of PXRD during the investigation of HCl concentration: (**a**) PXRD; (**b**) PDF; (**c**) G_NNN_; (**d**) PCA (The quantity of nilotinib and pH value were fixed at 0.5 g and 11.70, respectively).

As depicted in Figure 6a, the formation of an amorphous form was observed across all concentrations of HCl. However, it was revealed in Figure 6b that the samples synthesized at lower HCl concentrations had greater variability in the PDF traces and displayed a more prominent NNN peak compared to those produced at higher HCl concentrations. The intensity of the NNN peak further indicated that varying HCl concentrations significantly influenced the peak intensity of the samples, reflecting differing degrees of structural disorder. Consequently, an increase in HCl concentration correlated with a decrease in NNN peak height, suggesting the emergence of a more ordered amorphous solid structure, albeit characterized by a higher level of disorder relative to the more disordered structures observed at lower HCl concentrations. Furthermore, as illustrated in Figure 6c, the G_NNN_ value for the sample produced with a higher concentration of HCl exceeded that of samples synthesized with lower concentrations. Notably, the sample prepared with 2.0 mol/L HCl exhibited the lowest G_NNN_ value, signifying the highest degree of disorder. Consequently, 2.0 mol/L HCl was selected for further investigation in this study. Figure 6d demonstrated that in contrast to crystal Form A, the PCA score plot categorized all samples into two distinct groups: one corresponding to crystal Form A and the other to amorphous samples. Furthermore, the sample synthesized with 2.0 mol/L HCl was significantly separated from crystal Form A in the PC1 score plot, indicating a notable structural divergence from this reference form.

#### 2.3.2. R_c_ Analysis of DSC

**Figure 7:**
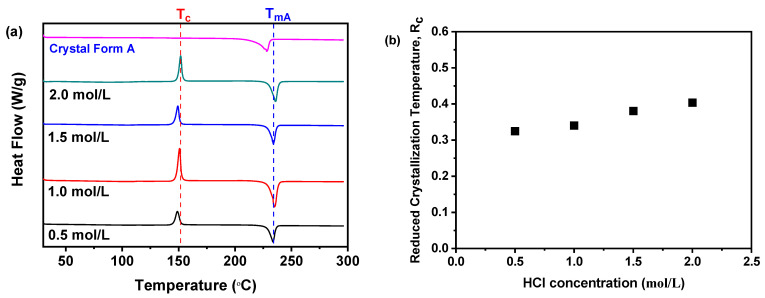
R_c_ analysis of DSC during the investigation of HCl concentration: (**a**) DSC; (**b**) R_c_ (The quantity of nilotinib and pH value were fixed at 0.5 g and 11.70, respectively).

As revealed in Figure 7a, a distinct endothermic peak was observed at 235 °C in all samples synthesized using varying concentrations of HCl, corresponding to the T_mA_, while an exothermic peak observed at approximately 150 °C indicates a transition from the amorphous to the crystal form. Furthermore, Figure 7b illustrates that the R_c_ values were influenced by the concentration of HCl. Specifically, the sample synthesized with a HCl concentration of 2.0 mol/L exhibited the highest R_c_ value. Conversely, as the HCl concentration decreased from 2.0 to 0.5 mol/L, the R_c_ value decreased, indicating that lower HCl concentrations are associated with a gradual decline in physical stability. Consequently, a concentration of 2.0 mol/L HCl was identified as the optimal condition in this study during the investigation of pH value.

### 2.4. Multivariate Function Analysis

To optimize the neutralization precipitation conditions for obtaining nilotinib in its amorphous form with high physical stability, PCA was utilized to assess the PDF data of samples prepared under different precipitation conditions. The experimental results of this analysis are summarized in Table 1.

**Figure 8:**
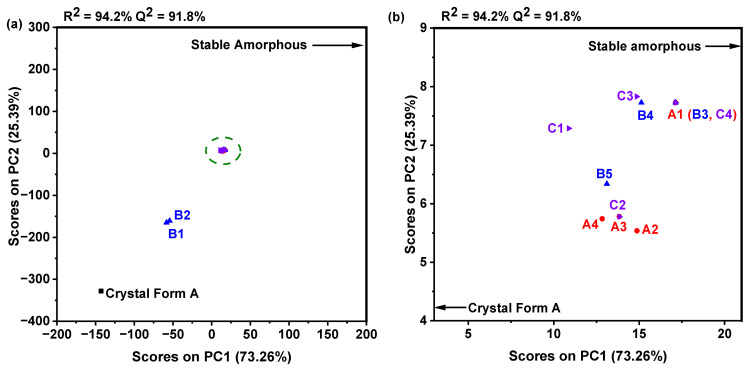
(**a**) PCA. (**b**) PCA, with an enlarged section of the circled area is in (**a**).

A PCA was performed using data obtained from PDF datasets, as illustrated in Figure 8a. This analysis effectively categorized the samples into three separate clusters. The first cluster comprised the crystal Form A, which served as the reference sample. The second cluster included samples collected at pH values of 1.10 and 2.65, which correspond to nilotinib hydrochloride monohydrate, designated as B1 and B2, respectively. The third cluster comprised other samples that indicated an amorphous form. Notably, the PCA plot effectively discriminated between the samples in the second cluster and those in the third cluster. Furthermore, the samples categorized within the third cluster, delineated by the circled region in Figure 8a, have been enlarged and illustrated in Figure 8b. It was also observed that the sample exhibiting the most significant deviation from the reference sample was A1 (alternatively labeled as B3 or C4), which was synthesized using 0.5 g of nilotinib, a pH of 11.70, and a HCl concentration of 2.0 mol/L. The results suggest that A1 (B3, C4) exhibits the most significant alterations in solid structure, indicating a greater level of disorder in comparison to both the reference sample and the other samples analyzed. Therefore, the experimental results imply that the optimal neutralization precipitation conditions for achieving the most physically stable amorphous solid were established with a nilotinib quantity of 0.5 g, a pH of 11.70, and a HCl concentration of 2.0 mol/L, despite this condition yielding a sample with the highest structural disorder among the amorphous solids analyzed.

### 2.5. Accelerated Stability Test Results

**Figure 9:**
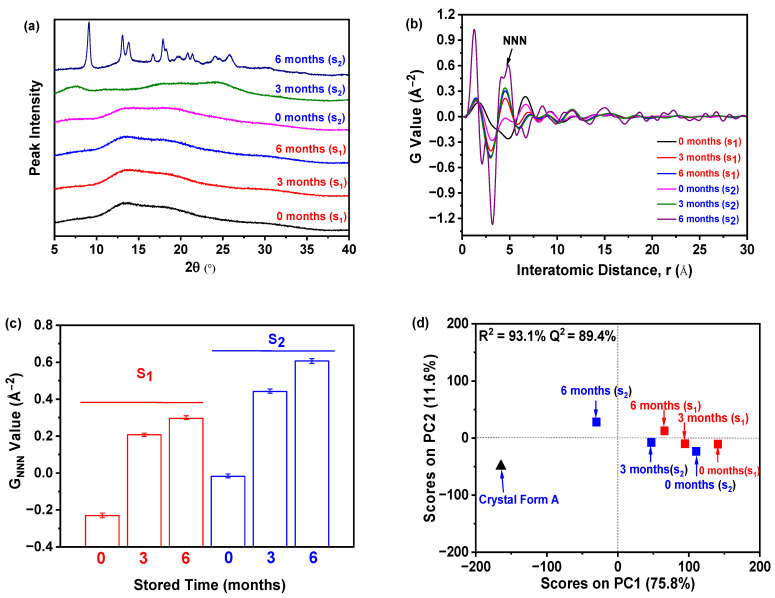
Accelerated stability test: (**a**) PXRD; (**b**) PDF; (**c**) G_NNN_; (**d**) PCA. (0 months (S), 3 months (S), and 6 months (S) represent the sample S stored for 0, 3, and 6 months, respectively. Subscripts 1 and 2 represent the samples S_1_ and S_2_).

As shown in Figure 9a, sample S_1_, which contained 0.5 g of nilotinib and underwent an accelerated stability test for a duration of 6 months, remained in an amorphous state. In contrast, sample S_2_, comprising 12 g of nilotinib and stored for periods of 0 and 3 months, also retained its amorphous characteristics initially; however, it transitioned to crystal Form A after 6 months, as evidenced by the PXRD patterns [40,41]. Consequently, S_1_ demonstrated enhanced physical stability relative to S_2_, a finding corroborated by the PDF and R_c_ metrics discussed in Section 2.1. The differentiation of amorphous forms using PXRD alone proved to be challenging, which necessitated the incorporation of PDF analysis (Figure 9b). Notably, sample S_2_, after 6 months of storage, exhibited considerable fluctuations and recorded the highest peak height for the NNN, while both S_1_ and S_2_ displayed an increase in NNN peak heights over the 6-month storage period.

Furthermore, the assessment of disorder within the samples was conducted through the analysis of the G_NNN_ value, as demonstrated in Figure 9c. Samples that were stored for a duration of 6 months demonstrated elevated G_NNN_ values in comparison to those stored for 0 and 3 months, suggesting that a lower G_NNN_ is correlated with an increase in disorder. It is noteworthy that the sample stored for 0 months exhibited the lowest G_NNN_, which aligns with a higher level of disorder. Additionally, the S_1_ sample stored for 0 months presented a lower G_NNN_ than the S_2_ sample, indicating a greater degree of disorder and physical stability in S_1_. Consequently, sample S_2_ demonstrated a greater propensity for crystallization relative to S_1_, as depicted in Figure 9a. Furthermore, as presented in Figure 9d, the samples were categorized the samples into three distinct groups: (1) crystal Form A (reference), (2) S_2_ after 6 months, and (3) other samples. The 6-month S_2_ sample was notably distinct along the PC1, resembling crystal Form A, whereas S_1_ at 0 months exhibited the greatest deviation, indicating a unique structural configuration and significant disorder. Despite both samples being stored for the same duration, S_1_ demonstrated superior stability compared to S_2_, thereby reinforcing the conclusion regarding the enhanced physical stability of S_1_.

In summary, the findings of this study indicate that a 6-month waiting period for the evaluation of physical stability using PXRD is unwarranted. Preliminary PXRD analyses, when enhanced by PDF and PCA, provide a timely and effective approach for evaluating disorder and physical stability in amorphous pharmaceutical compounds. Therefore, the combination of PXRD, PDF, and PCA methodologies presents significant advantages over conventional accelerated stability testing, enabling a more precise and efficient assessment of amorphous drug formulations.

### 2.6. Discussion on Future Work

In the realm of pharmaceutical manufacturing, the enhancement of production output is intrinsically linked not only to the economic advantages for the company but also to the reliability of drug supply and the therapeutic requirements of patients. Our current research indicates that while a more physically stable amorphous form of nilotinib can be precipitated with a reduced quantity, the practical application of this finding in industrial production settings presents significant challenges.

Upon conducting a more detailed analysis, we identified that the primary constraint arises from discrepancies between experimental methodologies and real-world operational practices. Specifically, during our investigation into the influence of the quantity of nilotinib on amorphous stability, we employed a relatively low stirring speed, which may have resulted in inadequate and inconsistent mixing of the two solutions during the neutralization process. Furthermore, the limited dimensions of the Buchner funnel utilized in the filtration phase constrained the filtration rate, thereby increasing the likelihood of complications associated with a higher quantity of nilotinib during large-scale production, such as crystallization and uneven particle distribution, which ultimately compromise the stability of the amorphous form.

In response to these findings, our forthcoming research will prioritize strategies to ensure the stability of the amorphous form of nilotinib while scaling up production, focusing on the precise regulation of process parameters. Specifically, we will aim to optimize stirring speed techniques to enhance production efficiency while ensuring uniform solution mixing. Additionally, we will investigate improvements to the filtration process, such as enlarging the Buchner funnel or employing more advanced filtration equipment, to expedite the filtration rate and mitigate instability factors associated with prolonged filtration. By implementing these strategies, we aspire to significantly improve the preparation efficiency and yield of the amorphous form of nilotinib while maintaining product quality, thereby better addressing market demands and serving a diverse patient population.

## 3. Materials and Methods

### 3.1. Materials

**Figure 10:**
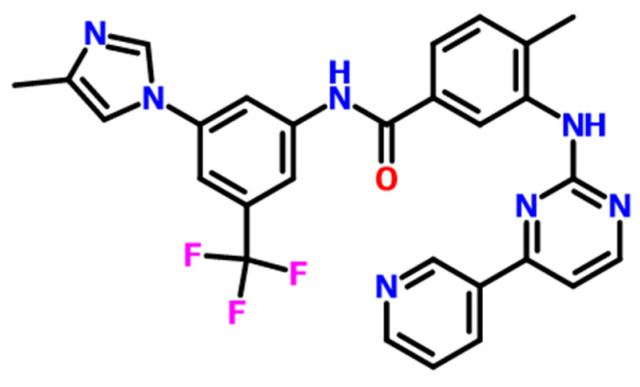
The molecular structure of nilotinib.

Anhui Heryi Pharmaceutical Co., Ltd. (Tianchang, China) provides nilotinib in solid form with a purity of 98% (Form A), and its molecular structure is illustrated in Figure 10. HCl and NaOH, both with a purity of 99%, were sourced from Titan Technology Co., Ltd. (Shanghai, China). 

### 3.2. Methods

#### 3.2.1. Neutralization Precipitation

**Figure 11:**
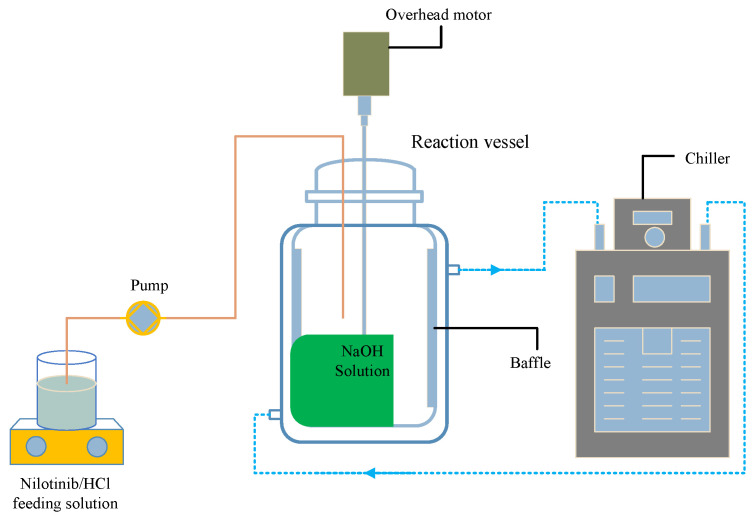
Experimental configuration for the neutralization precipitation of amorphous nilotinib.

As depicted in Figure 11, a comprehensive study was conducted to assess the quantity of nilotinib by fully dissolving solid nilotinib in various amounts (0.5, 3, 6, and 12 g) within HCl solutions of a constant concentration of 2.0 mol/L, utilizing corresponding volumes of 7.5, 45, 90, and 180 mL. This procedure yielded nilotinib hydrochloride solutions with a uniform concentration of 0.126 mol/L. In a distinct experimental setup aimed at evaluating HCl concentration, solutions were prepared across a spectrum of concentrations (0.5, 1.0, 1.5, and 2.0 mol/L). The dissolution was performed at a controlled temperature of 25 °C, followed by filtration to eliminate any remaining solid impurities. Subsequently, an equivalent volume of NaOH solution, matching the concentration of the initial HCl solution, was introduced into a baffled reactor (S300, Beijing Century Senlang Experimental Instrument Co., Ltd., Beijing, China). During the pH assessment, a fixed volume of 7.5 mL of nilotinib hydrochloride solution (prepared with 2.0 mol/L HCl) was employed, while the volume of 2.0 mol/L NaOH was systematically varied (6.5, 7.0, 7.5, 8.0, and 8.5 mL), resulting in corresponding pH values of 1.01, 2.65, 11.70, 12.54, and 13.17. During the experiment, the stirring speed was consistently maintained at 500 rpm as the nilotinib hydrochloride solution was gradually introduced into the NaOH solution within the reactor. This addition was facilitated by a peristaltic pump (model BT100FC, Baoding Rongbai Constant Flow Pump Manufacturing Co., Ltd., Baoding, China) operating at a constant flow rate of 5.0 mL/min, thereby initiating the neutralization precipitation. Due to nilotinib’s low aqueous solubility in free base form, rapid solid suspension formation occurred upon mixing nilotinib hydrochloride with NaOH solution. The suspension was stirred and aged for 5 min post-mixing. The wet filter cake was isolated using a circulating water vacuum pump (SHZ-III A, Gongyi Ruide Instrument Equipment Co., Ltd., Gongyi, China), rinsed with 50 mL of deionized water, and dried in a vacuum oven (DHG-9055A, Wujiang Yonglian Machinery Equipment Factory, Wujiang, China) at 40 °C for 18 h. The samples were stored in capped glass bottles at room temperature until further analysis.

#### 3.2.2. Filter Test

The sample suspension underwent filtration utilizing a vacuum pump (SHZ-III A) in conjunction with a 6 cm Buchner funnel (Shanghai Titan Technology Co., Ltd., Shanghai, China). The duration of the filtration process was meticulously recorded until the cessation of filtrate flow was observed. The filtration rate was determined by calculating the ratio of the suspension volume to the filtration time.

#### 3.2.3. Characterization of Solid Samples

The solid samples that were obtained were characterized using PXRD, DSC, and FBRM. Detailed information about these characterization methods is available in our earlier research [18,19,20,22].

#### 3.2.4. Pair Distribution Function (PDF)

The examination of the PDF was conducted through Fourier transformation of PXRD data, utilizing the PDFgetX2 software (version 1.0). Detailed configuration parameters are provided in reference [53].

#### 3.2.5. Principal Components Analysis (PCA)

PCA was employed to analyze variations in PXRD and PDF data across solid samples. Data preprocessing and scaling were performed using SIMCA 15, following the methodology of Karmwar et al. [54]. PCA focused on interatomic distances from 0 to 15 Å, encompassing principal peaks characteristic of amorphous solids [34,55].

#### 3.2.6. Accelerated Stability Test

Nilotinib amorphous samples that precipitated at 0.5 and 12 g, respectively, designated as S_1_ (0.5 g) and S_2_ (12 g), were selected for an accelerated stability assessment. Following a drying process, each sample was subdivided into three portions. One portion from each sample (0 (S_1_) and 0 (S_2_)) was subjected to immediate analysis using PXRD. The remaining portions were stored in uncapped glass containers and stored in a drug stability testing chamber (YP-250GSP, Beijing Hengtai Fengke Testing Equipment Co., Ltd., Beijing, China) at a temperature of 40 °C and relative humidity of 75% for durations of 3 and 6 months, labeled as 3 (S_1_), 6 (S_1_), 3 (S_2_), and 6 (S_2_). After the storage period, data were collected using PXRD, PDF, and PCA.

## 4. Conclusions

The formulation of amorphous solid pharmaceuticals presents significant challenges, primarily due to their intrinsic tendency for physical instability. This instability may result in unintentional crystallization, which subsequently diminishes solubility and lowers dissolution rates, ultimately undermining the overall performance and effectiveness of these pharmaceutical products. This research was carefully crafted to develop a physically stable amorphous formulation of nilotinib by fine-tuning key parameters in the neutralization precipitation process. The parameters examined included the quantity of nilotinib, the pH value, and the concentration of HCl. The results indicated that the ideal formulation consisted of 0.5 g of nilotinib, a pH of 11.70, and 7.5 mL of HCl with a concentration of 2.0 mol/L, leading to amorphous samples with improved physical stability. It is essential to acknowledge that the current observation is limited to the specific conditions of the experimental setup employed and may not necessarily apply to scaled-up experiments. The parameters, variables, and environmental factors that are rigorously controlled in this particular configuration may vary significantly when the experiment is expanded, potentially resulting in different outcomes. Consequently, while the findings of the present study offer valuable insights, it is crucial to approach any extrapolation with caution, recognizing that the behaviors observed may not be replicated in a scaled-up experimental context. This highlights the necessity for further research under diverse conditions to achieve a comprehensive understanding of the phenomenon and to ensure the validity and applicability of the results across various scales and settings. Additionally, the study introduced innovative methods for evaluating physical stability, employing PDF and PCA on PXRD data, along with R_c_ values derived from DSC data. These methodological advancements represent a significant improvement in the evaluation of physical stability, facilitating a more rapid assessment while yielding results that are more precise and accurate than those derived from traditional accelerated stability testing methods.

## Figures and Tables

**Table 1:** The criteria for the precipitation of amorphous solids and their corresponding nomenclature.

Experiments	Fixed Precipitation Conditions	Variables	Notation
Quantity of Nilotinib (g)	pH value: 11.70 HCl concentration: 2.0 mol/L	0.5	A1
		3	A2
		6	A3
		12	A4
pH Value	Quantity of nilotinib: 0.5 g HCl concentration: 2.0 mol/L	1.10	B1
		2.65	B2
		11.70	B3 (A1)
		12.45	B4
		13.17	B5
HCl Concentration (mol/L)	Quantity of nilotinib: 0.5 g pH value: 11.70	0.5	C1
		1.0	C2
		1.5	C3
		2.0	C4 (A1)
Reference Sample	/	/	Crystal Form A

## Data Availability

Data are contained within the article.
